# Bridging Between Japan-Originated Inorganic Chemistry Theories and the Latest DFT Calculations

**DOI:** 10.3390/molecules30143010

**Published:** 2025-07-18

**Authors:** Takashiro Akitsu

**Affiliations:** Department of Chemistry, Faculty of Science, Tokyo University of Science, 1-3 Kagurazaka, Shinjuku-ku, Tokyo 162-8601, Japan; akitsu2@rs.tus.ac.jp

There are two concepts that were discovered by Japanese researchers that appear in most inorganic chemistry textbooks: the spectrochemical series [1] and the Tanabe–Sugano diagram [2]. The spectrochemical series was proposed by Ryutaro Tsuchida of Osaka University. It experimentally determined the order in which the wavelengths of the absorption spectrum bands shift when the ligands of a hexacoordinate octahedral cobalt (III) complex are systematically substituted. With the establishment of the ligand field theory [3], this came to be theoretically explained in the form we know today as the order of ligand field strength and the magnitude of ligand field splitting. The “angular overlap model (AOM)” [4], which goes a little further than the ligand field theory, allows us to discuss σ- and π-bonds in coordination bonds (AOM *e*_σ_ and *e*_π_ parameters), as well as their donor and acceptor properties.

The Tanabe–Sugano diagram is a result of theoretical physics and is located at the intersection of coordination chemistry and solid-state physics. It quantitatively illustrates the energy of the terms due to the ligand field splitting of a hexacoordinate octahedral metal complex according to the strength of the ligand field, taking into account the repulsion between electrons. It can also explain why the gemstone ruby is red, the basic electronic state of iron in hemoglobin contained in blood, and is a good example showing the relationship between coordination chemistry and ligand field theory and earth sciences and biology.

As reviewed above [5], I have been studying the structure and electronic state of metal complexes for about 30 years. Nowadays, de facto standard density functional theory (DFT) calculation programs [6] and inexpensive, high-performance computers (sometimes even a laptop computer may be sufficient) are widely available in laboratories. As someone who has studied group theory and quantum mechanics and has been researching experimental interpretations using empirical theoretical models, I am concerned about the relationship with recent computational chemistry. It all started when, while comparing the electron density of coordinate bonds obtained from X-ray crystal structure analysis with DFT calculations, it became obvious that it was impossible to simply simulate experimental data with intense thermal vibrations and large temperature factors (low electron density) [7,8].

For example, “ligand field strength” is treated as the effect of σ- and π-bonds on d orbitals of coordinate bonds, but DFT does not give “local” calculation results. In carbonyl complexes, what does π- back donation increasing the ligand field splitting (Δ_o_) mean? The “Racah parameters (*A*, *B* and *C*)”, which express the energy of ground and excited terms in multi-electron systems, are integral values that express the repulsion between electrons (originally based on atomic spectra). When changing the “ligand field strength” in DFT, does the spin multiplicity of the ground term change? (It is possible to set this during input and compare the energy stability.). Which orbital transition around HOMO-LUMO will the “excited term” in the *O*_h_ point group represent? The nephelauxetic effect (*B*_0_/*B*) is an indicator of covalent bonding according to the literature on inorganic chemistry. With the aid of the latest research [9], where in the DFT output file should we look for a clue after UV-vis measurements?

The electronic state of a metal complex contains information on multiple physical properties, such as the ground state (magnetism and electron spin), excited state (optical transition), and lowest unoccupied molecular orbital (reduction potential). Is it possible to interpret and predict the magnetic anisotropy parameters (*D* and *E*) [10], which determine the potential energy of a single-molecule magnet [11], not only from magnetic circular dichroism [12] or electron spin resonance experiments and alternating current magnetic susceptibility measurements, but also from DFT? In light of this need, I hope to establish correspondence with the current framework.
